# Personalized Trial Ethics and Institutional Review Board Submissions

**DOI:** 10.1162/99608f92.2ded0fc5

**Published:** 2022-09-08

**Authors:** Joyce P. Samuel, Susan H. Wootton

**Affiliations:** 1 John P. and Kathrine G. McGovern Medical School, Health Science Center, University of Texas, Houston, Texas, United States of America

**Keywords:** ethics, consent, personalized trial, N-of-1 trial

## Abstract

The ethical and regulatory oversight of any clinical activity related to human subjects is commonly determined based on its categorization as either clinical practice or research. Prominent bioethicists have criticized the traditional distinctions used to delineate these categories, calling them counterproductive and outmoded, and arguing that learning and clinical practice should be deliberately and appropriately integrated. Personalized trials represent a clinical activity with characteristics that overlap both categories, making ethical and regulatory oversight requirements less straightforward. When the primary intent of the personalized trial is to assist in the conduct of individualized patient care with an emphasis on protecting the clinical decision from the biases inherent in usual clinical practice, how should this activity be regulated? In this article, we will explore the ethical underpinnings of personalized trials and propose various approaches to meeting regulatory requirements. Instead of imposing standard research regulations on the conduct of all personalized trials, we recommend that personalized trialists and IRB panels should consider whether participation in a personalized trial results in any foreseeable incremental increase in risk to the participant compared with usual care. This approach may reduce regulatory barriers, which could promote more widespread uptake of personalized trials.

## 1. Introduction

Consider the following hypothetical clinical scenario: Dr. Brown is contemplating the use of amitriptyline, a tricyclic antidepressant (TCA) medication to treat chronic abdominal symptoms in a patient with irritable bowel syndrome (IBS). The patient is eager to find a solution to the symptoms that have disrupted his work productivity and quality of life, but is wary of the potential side effects of the treatment. TCAs have been associated with various adverse effects, including fatigue, cardiac rhythm disturbances, palpitations, and lightheadedness. A significant placebo effect has been described in this population, and the patient is concerned that he could be exposed to the potential harms of the treatment without any true benefit. After a quick literature review, Dr. Brown finds conflicting recommendations in the clinical practice guidelines published by two major specialty societies, and randomized controlled trials raise concern that it may be difficult to predict which IBS patients may be responders versus nonresponders. Dr. Brown has encountered an all-too-common scenario in which her attempts to base clinical practice on evidence-based medicine are thwarted by conflicting or limited data.

The integration of clinical epidemiology, human subjects research, and evidence-based guidelines is the cornerstone of evidence-based medicine. At the foundation of evidence-based medicine lies the assumption that clinical practice informed by an understanding of the underlying clinical research evidence will result in superior patient care compared to practice that relies solely on intuition and clinical experience (Guyatt et al., 1992). However, evidence-based practice can be limited when high-quality evidence is lacking. Human subjects research is integral to this paradigm, and standards to protect human subjects have evolved over recent decades.

## 2. History of Research Oversight and Regulations

Regulations protecting human subjects in the United States became effective in 1974 with the passage of the National Research Act that established the National Commission for the Protection of Human Subjects of Biomedical and Behavior Research for the United States Department of Health, Education, and Welfare (HEW). The commission set forth the basic ethical principles that should underlie the conduct of biomedical and behavioral research involving human subjects in the seminal paper, *The Belmont Report* (n.d.-a). The report outlined primary principles underlying ethical research involving human subjects that include beneficence, justice, and respect for persons. The principle of beneficence in clinical research obliges researchers to maximize the benefits of participation and reduce the risk of harm. Justice in clinical research emphasizes the importance of fairness in distribution of both the benefits of research and its burdens. Respect for persons involves allowing individuals to be autonomous and protecting those with diminished autonomy, whether due to immaturity or incapacitation. The methods used to recognize these principles are informed consent, risk/benefit analysis, and appropriate selection of patients. *The Belmont Report* has been the most influential of all U.S. ethics and bioethics commissions on U.S. public policy and federal regulation (Beauchamp, 2020).

In addition to establishing the Belmont Commission for the DHEW, the (n.d.-b) established guidelines for research with human subjects. These regulations introduced the concept of the institutional review board (IRB) to the process of research funded by the HEW. In 1991, the regulation of the HEW—now the Department of Health and Human Services (HHS)—became the Common Rule for 16 federal agencies (Protection for Human Research Subjects, 2022).

Within the Common Rule, various definitions were set forth, including one for research, which is defined as the “systematic investigation, including research development, testing, and evaluation, designed to develop or contribute to generalizable knowledge,” and practice “interventions that are solely designed to enhance the well-being of an individual patient or client and have a reasonable expectation of success.” *The Belmont Report* called for distinguishing clinical practice from clinical research. Practice referred to the “interventions that are solely designed to enhance the well-being of an individual patient or client and have a reasonable expectation of success,” whereas research designated “an activity designed to test a hypothesis, permit conclusions to be drawn and thereby to develop or contribute to generalizable knowledge.”

## 3. Experimentation in Clinical Practice

In our previous example, in usual clinical practice Dr. Brown may decide to try amitriptyline in the patient with irritable bowel syndrome. After a few weeks the patient returns for a follow-up visit to evaluate the response and determine the next course of action. As Dr. Brown considers whether a dose adjustment is warranted or whether the treatment should be abandoned for another option, she is free to use her judgment about which symptoms to ask about, and what threshold of symptom reduction to characterize as treatment success versus failure. The clinician would be unlikely to differentiate between placebo effect and true medication effect. As Dr. Brown practices the art of medicine, one would expect her decisions to vary from patient to patient; however, this variability is likely affected by more than simply heterogeneity in treatment effects. Her clinical decisions could be viewed as haphazard, affected by a variety of factors, including her own baseline skepticism or optimism about the likelihood of treatment success, her anecdotal recall of treatment response in her previous patients, or the frustration level of the patient. As long as she has no intention of publishing her findings so that others can learn from their experiences, she may treat her patients with no regulatory oversight at all. She is not obligated to prospectively formulate her treatment plan nor submit her treatment plan to an external review board.

Even when clinicians diligently attempt to make evidence-informed decisions, present-day clinical practice remains polluted with many sources of bias. These may include cognitive bias, patient reluctance to tell their physician that a treatment is not working, misclassifying unrelated symptoms as side effects from a treatment, and other factors that may obscure the true treatment effect. One approach to minimize these biases from decision-making in clinical practice is to employ personalized trials, in which the patient and the clinician mutually agree to undergo a single patient (N-of-1) trial, also known as a personalized trial. In a personalized trial, a single patient undergoes a within-subject crossover trial of alternating blocks of various treatment options until the optimal therapy is identified. Treatment blocks can include treatment versus placebo or versus other active treatment. Personalized trials borrow bias-minimizing techniques from clinical research paradigms to produce the most accurate estimate of treatment effects for the individual. The systematic nature of personalized trial methodology should prompt the consideration of personalized trials to be an elevated form of patient care, in which potential adverse effects from the treatment are more carefully queried than in usual care, and treatment efficacy is scrutinized more systematically.

Dr. Brown recognizes that a personalized trial may allow her to objectively understand the effects of amitriptyline in her patient. By using an identical placebo to facilitate blinding of both herself and the patient, testing the treatments in randomized order, and using a validated outcome measurement tool to assess treatment effects at the end of each treatment period, she could gather enough data from her patient to more confidently make a less-biased decision about whether this treatment works in her patient. Her approach could be considered risk-reducing compared to usual care, as she is more carefully assessing for side effects and more rigorous in her judgments of treatment success or failure.

Since she is repeatedly faced with this same clinical uncertainty in her other patients with irritable bowel syndrome, she decides to use this same approach in other patients with this disorder. If she protocolizes her approach and conducts similar personalized trials in a series of similar patients, she could produce generalizable knowledge that could be of great value to her colleagues and counterparts in other institutions. However, although her first priority is to improve the well-being of her patients, because she has protocolized her approach, employed randomization (instead of ‘haphazardization’) and intends to publish her results in a scientific journal, many institutions and journal editors would deem this to be clinical research, requiring IRB oversight and written documentation of informed consent of all participants. The debate about the research ethics of personalized trials has been discussed and debated for as long as this approach has been used in clinical medicine (n.d.-c).

The application of *The Belmont Report* four decades after its original publication has become increasingly challenging due to the significant changes that have occurred within the context of biomedical research (Friesen et al., 2017)(Wootton et al., 2013). Quality improvement (QI) activities offer an example of where the boundary between research and practice have become vague. QI is defined as the “systematic data-driven guided activities designed to bring about immediate improvement in health delivery in particular settings” (Baily et al., 2006). QI activities are important data-driven components of hospital operations that involve human participants and learning from experience.

Institutions throughout the United States have proposed criteria to determine when IRB approval of QI activities is needed. Examples of such criteria include when the QI activity 1) seeks to develop new knowledge or validate new treatments rather than to assess the implementation of existing knowledge, 2) uses methodology that employs a standard research design such as randomization, 3) involves funding from outside the organization, 4) involves a delay in the implementation, or 5) when the risks from the intervention to participants are greater than minimal. Criteria are variable and speak to challenges in application regulation for local IRBs. Personalized trial protocols that meet the definition of a QI activity could be considered outside the purview of the IRB depending on the specific treatments being tested and the incremental risk (additional relative to usual care) to participants (Samuel et al., 2016).

When personalized trials are used to compare two or more active treatments, one could classify them as a form of comparative effectiveness research (CER). The Institute of Medicine defines CER as the generation and synthesis of evidence that compares the benefits and harms of alternative methods to diagnose, treat, and monitor or improve the delivery of care. The purpose of CER is to assist consumers, clinicians, purchasers, and policymakers to make informed decisions at both the individual and population levels. In 2014, several prominent bioethicists including Ruth Faden, Tom Beauchamp (the author of *The Belmont Report*), and Nancy Kass called for streamlining both consent requirements and oversight practices for CER, provided these studies are grounded within an ethically robust learning health care system (Faden et al., 2014). In studies comparing low-risk and commonly used treatment options that are not considerably different in ways that matter to patients, it could be sufficient to simply inform patients of the study and provide them with an opportunity to decline participation. The moral foundation for a mature learning health care system is outlined in a proposed ethics framework published by Faden and colleagues (Faden et al., 2013).

## 4. Approaches to Consent

Respect for patient autonomy and safeguarding a patient’s trust in medical practice are some of the more commonly cited reasons justifying the need for informed-consent processes in clinical research and clinical practice (Levine, 1983). The conduct and documentation of informed consent vary widely between clinical research and practice. In clinical research, consent processes are formal and highly regulated. All elements of the consent are incorporated into a written document preapproved by an IRB, which must be signed by the participant and stored by the investigator.

However, in clinical practice the process is much less formal and mostly unregulated. In clinical practice, a discussion of the risks, benefits, and opportunity to make a choice about treatment represents good clinical practice, however, this discussion may be informal (not written), and verbal assent may be considered sufficient in many cases (Egan, 2008). In the case of invasive procedures or surgeries, written consent is commonly obtained, however, the specific elements included in the discussion or written consent form may not be externally reviewed prior to implementation.

The disparities in these two approaches can be attributed to major differences in the investigator–subject relationship versus the physician–patient relationship, which may be viewed on opposite ends of a spectrum. The investigator usually has a brief relationship with the subject, which is only long enough to accomplish a single purpose—to enlist the patient to participate in a project that is meant to serve the interests of society, interests that may be in conflict with those of the subject. In contrast, the physician–patient relationship may be long-lasting, with the primary objective of improving the well-being of the patient and meeting their unique needs.

Where does the personalized trial fall on this spectrum? If a physician–patient relationship has been developed over time, then does the addition of bias-reducing techniques to the decision-making approach fundamentally change the relationship such that the consent process should move closer to that which is required for research? If certain conditions are met, a less formal consent process similar to what is acceptable for usual clinical practice could also be sufficient with a personalized trial. We propose the following conditions:

1. The primary underlying motivation for the personalized trial remains the same as in usual clinical practice: to provide the best possible care for the patient and to meet the patient’s goals of care. A major goal of informed consent in clinical research is to provide participants with key information that allows them to understand the tradeoffs in participating, including the risks of harm as well as the possibility of no direct personal benefit in some cases, such as a placebo-controlled parallel group trial in which the participant could be randomized to placebo (n.d.-d). However, in the case of personalized trials, the patient’s well-being remains the primary motivation of the endeavor.
2. There is no significant incremental increase in the risk of harm to the patient over and above that which is expected in usual practice. Some risk of harm is nearly ubiquitous in usual clinical practice. When clinicians use an informal trial of therapy, the patient is exposed to the potential risks of all the therapies to be tested. If the same therapies are tested using a formalized process within a personalized trial protocol, then only the incremental increase in the risk of harm compared to usual care should be considered when deciding which consent approach is most appropriate (McKinney et al., 2015). The interpretation of ‘minimal risk’ to refer to the incremental risk added by study participation is made clear in the Common Rule, “In evaluating risks and benefits, the IRB should consider only those risks and benefits that may result from the research (as distinguished from risks and benefits of therapies subjects would receive even if not participating in the research)” (n.d.-e).
3. The patient understands the process and agrees to participate. Personalized trials cannot practically be conducted without agreement from the patient. As Gordon (n.d.-f) described in “A Clinician’s Guide for Conducting Randomized Trials in Individual Patients,” a personalized trial should be done only with an enthusiastic patient who understands the experiment, and the trial is by nature “a cooperative venture between clinician and patient” (p. 499). Patients should be given the opportunity to make a sound decision about whether to participate.
4. The personalized-trialist is a clinician-scientist with training in human-subjects protection and research ethics. Clinicians without such training may require additional oversight to ensure the principles of autonomy, disclosure, and understanding are maintained.

(Dickert et al., 2017) argue that the informed consent process in clinical research serves several distinct functions, including 1) providing transparency, 2) preserving patient autonomy, 3) promoting concordance with patient’s values, 4) protecting patient welfare, 5), promoting trust, 6) meeting regulatory requirements, and 7) promoting integrity in research. If the consent process is viewed through solely a legalistic or regulatory lens, the written consent form in clinical research may devolve to serve primarily as an instrument to protect the interests of the investigators and their institutions, safeguarding them from civil or criminal liability. In the context of a personalized trial, a careful conversation with the physician and provision of clear and succinct written materials may impart to the patient a greater understanding and deeper trust in the personalized trial process compared to a lengthy and legalistic signed consent form (Tam et al., 2015).

## 5. Institutional Review Board Oversight

The views of IRB members on whether personalized trials require IRB approval and whether they meet the federal definition of human subjects research according to the Common Rule (n.d.-g) varies considerably. A survey of members of IRBs accredited by the Association for the Accreditation of Human Research Protection Programs found that 43% of respondents viewed N-of-1 trials as research, however, an additional 32% reported that they did not know or it depended on a case-by-case basis (Cen et al., 2016). Among the 12 respondents (21%) who reported having an existing IRB policy on N-of-1 trials, 100% of these policies considered N-of-1 trials as *not* meeting the federal definition of human subjects research. A systematic review of personalized trials in 2011 found that among 108 unique protocols described in the literature, 69% reported that IRB approval was sought and granted (Gabler et al., 2011). When IRB panels are unfamiliar with personalized trial study design, this could result in the imposition of requirements that may not be warranted. The establishment of IRB policies about personalized trials could help reduce undue regulatory burdens and promote increased uptake of these activities.

At the University of Texas Health Science Center at Houston (UTH), efforts are being made to advance a learning health care system where research and other learning activities can be more readily embedded into clinical practice. At UTH we conducted a series of personalized trials in adolescents with primary hypertension to identify the preferred pharmacologic treatment for the individual patient (Samuel et al., 2019). Patients completed a crossover trial of three active treatment options for 2-week blocks, with treatments given in random order and treatment effects measured with a 24-hour ambulatory blood pressure monitor at the end of each treatment period. The blood pressure change in each treatment block was assessed by an outcome assessor blinded to drug name, and the preferred treatment for the individual was defined as that which produced the greatest blood pressure reduction without intolerable patient- or parent-reported side effects. The protocol was submitted to the IRB as a QI activity, and after preliminary review the IRB agreed to classify it as such given that all of the treatments to be tested are commonly used in clinical practice and the project sought to improve the decision-making process of how the treatment was selected for the individual patient. The conduct thereafter was not regulated by the IRB and we proceeded with verbal consent from the patient and parent along with provision of a written description of the study procedures. In a separate study, we are currently completing a randomized clinical trial in which children with hypertension were randomized to either a personalized trial using a similar protocol described above or to usual care, in which treatment is selected based on clinician preference. Due to the use of randomization to determine treatment assignment, the study was submitted to the IRB as a conventional human subjects research protocol. However, given the minimal risk of the protocol compared to usual care, a streamlined consent approach was approved. Verbal consent was obtained from the patient and parent and a single-page information sheet was provided, but written documentation of consent with signature was not required.

## 6. Conclusion

Personalized trials do not fit neatly into the usual categories used to delineate which activities require formalized informed consent or IRB oversight. We recommend that IRB panels should be educated in the personalized trial process so that policies can be developed to facilitate a simplified regulatory oversight process. As IRBs become more familiar with this methodology, their approach may evolve into a more nuanced examination of the specific risk–benefit analysis of the protocol under review and appropriate consent approach depending on the specific treatments, disease, and population to be included. This may begin to reduce the regulatory hurdles in the conduct of these trials, which could improve the ease of integration into clinical practice.
